# Comparative Computational Study of Interaction of C_60_-Fullerene and Tris-Malonyl-C_60_-Fullerene Isomers with Lipid Bilayer: Relation to Their Antioxidant Effect

**DOI:** 10.1371/journal.pone.0102487

**Published:** 2014-07-14

**Authors:** Marine E. Bozdaganyan, Philipp S. Orekhov, Alexey K. Shaytan, Konstantin V. Shaitan

**Affiliations:** Biological department, M.V. Lomonosov Moscow State University, Moscow, Russia; Instituto de Tecnologica Química e Biológica, UNL, Portugal

## Abstract

Oxidative stress induced by excessive production of reactive oxygen species (ROS) has been implicated in the etiology of many human diseases. It has been reported that fullerenes and some of their derivatives–carboxyfullerenes–exhibits a strong free radical scavenging capacity. The permeation of C_60_-fullerene and its amphiphilic derivatives–C_3_-tris-malonic-C_60_-fullerene (C_3_) and D_3_-tris-malonyl-C_60_-fullerene (D_3_)–through a lipid bilayer mimicking the eukaryotic cell membrane was studied using molecular dynamics (MD) simulations. The free energy profiles along the normal to the bilayer composed of 1,2-dipalmitoyl-sn-glycero-3-phosphocholine (DPPC) for C_60_, C_3_ and D_3_ were calculated. We found that C_60_ molecules alone or in clusters spontaneously translocate to the hydrophobic core of the membrane and stay inside the bilayer during the whole period of simulation time. The incorporation of cluster of fullerenes inside the bilayer changes properties of the bilayer and leads to its deformation. In simulations of the tris-malonic fullerenes we discovered that both isomers, C_3_ and D_3_, adsorb at the surface of the bilayer but only C_3_ tends to be buried in the area of the lipid headgroups forming hydrophobic contacts with the lipid tails. We hypothesize that such position has implications for ROS scavenging mechanism in the specific cell compartments.

## Introduction

**Figure 1:**
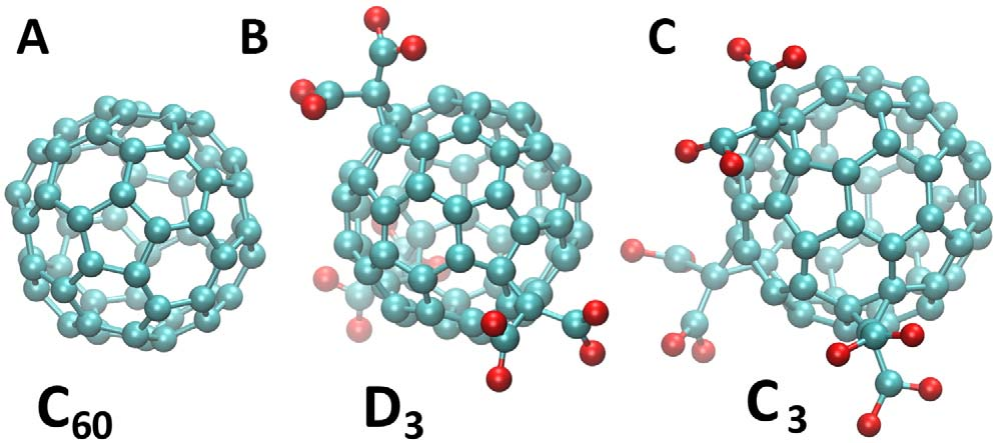
The studied species of the C_60_ fullerene. A. Buckminsterfullerene C_60_. B. D_3_ stereoisomer of tris-malonyl-C_60_-fullerene. C. C_3_ stereoisomer of tris-malonyl-C_60_-fullerene.

Fullerenes and their derivatives are considered perspective for various applications in medicine and pharmacology [1]. Particularly, C_60_-fullerene and tris-malonic C_60_-fullerene (Figure 1) are known as free radical scavengers, that have been shown to protect cells from free radicals (including ROS) that can induce apoptotic injuries *in vitro* [2], [3] as well as in different cell types: neuronal cells [4], hepatoma cells [5] and epithelial cells [6]. At the same time, some of fullerene derivatives (for example dendrofullerenes) [7] inhibit HIV-protease, what gives another prospect for their biomedical use. Harhaji et al. demonstrated [8] the antitumor effect of the water suspension of C_60_ on glioma cell cultures when irradiated by light: high concentrations of fullerene caused necrosis, while low concentrations stopped proliferation of the cells and eventually lead to autophagy.

Fumelli et al. [9] have demonstrated that carboxyfullerenes protected human keratinocytes from apoptosis induced by ultraviolet-B (UVB) irradiation. Later the possibilities of preventing the neurotoxicity generated by levodopa (L-3, 4-dihydroxyphenylalanine) have been demonstrated as chemical properties of a water-soluble fullerene derivative and ascorbic acid [10]. Wang et al. compared [11] antioxidant activity of the two regioisomeric forms of tris-malonic fullerene (Figure 1)–C_3_ (with the malonic groups localized on one side of the fullerene molecule) and D_3_ (with the malonic groups distributed evenly). It was found that C_3_ has a higher activity against ROS than D_3_, presumably due to its better interaction with biomembranes. However, the molecular basis of these interactions remains elusive. Particularly, the process of passive transport through cell membranes of C_60_ and its derivatives must be elucidated.

Most of the fullerenes get inside the cell by the energy-dependent mechanism of endocytosis [12]. On the other hand, hydrophobic C_60_ molecules can directly interact with the biomembrane and penetrate it in an energy-independent manner. One of the theoretical methods, which allows to tackle this problem, is computational simulation by molecular dynamics. These can be used to study the penetration of small molecules [13] as well as nanoparticles [14] through biomembranes. Several atomistic molecular dynamics simulation studies have been performed that investigate the interactions of C_60_ and its derivatives with model biological membranes [15], [16], [17]. It was shown that water-soluble fullerene derivatives do not penetrate into the membranes as the C_60_ does [16], [17], [18]. In [15] a coarse-grain model was applied to study penetration abilities of multiple fullerenes into different membranes and the authors showed that multiple fullerenes inside the bilayer cause no deformation of the membrane.

Here we report a comparative study of the interaction of C_60_-fullerene and its amphiphilic derivatives, C_3_ and D_3_, with eukaryotic membranes composed of DPPC lipids using molecular dynamics simulations. We performed series of unrestrained MD simulations and calculated free energy profiles as a function of the position of the studied molecules along the bilayer normal. The aim of our investigation is to explain the mechanism of ROS scavenging for fullerene species with different activities. We found out that molecules have different penetration depths into the bilayer what has implications regarding the possible antioxidant effects of C_60_ carboxyl derivatives.

## Materials and Methods

Four different systems were used for the simulations: a lipid bilayer with one molecule C_60_, with ten molecules of C_60_, with one C_3_ molecule and with one D_3_ molecule. The model of the eukaryotic membrane consisted of 128 solvated DPPC lipids. The C_60_-, C_3_- or D_3_-molecules were placed in water and did not contact the lipid surface in the beginning of the simulations. In all simulations we used the Berger model for lipids [19] and the SPC/E water model [20]. The force field parameters for C_60_, C_3_ and D_3_ were set as follows. The lengths of the two types of C-C bonds in fullerenes were set to 0,139 nm and 0,144 nm according to [21]. Parameters of the van der Waals interactions were taken from [16], [17]. The atomic partial charges of carbon atoms in a fullerene were set 0 for the all of them due to the symmetry of the molecule, while the Mulliken partial atomic charges for C_3_ and D_3_ derivatives were calculated in GAMESS suite (see SI, Table S1) using *ab initio* Hartree-Fock method with 6–31G** basis set (prefaced by the geometry optimization of the molecules at the same level of theory).

The all of MD simulations were carried out using Gromacs 4.5 [22]. Simulations were carried out in the NPT ensemble: semi-isotropic pressure coupling (Parrinnelo-Rahman barostat [23], time constant 2 ps) and constant temperature 323 K (Nose-Hoover thermostat [24], [25], time constant 2 ps). Lipids and water were coupled independently to the heat bath. Periodic boundary conditions were applied in all three dimensions. All bond lengths were kept constant using the LINCS [26] algorithm. The time step was 2 fs, long-range electrostatic interactions were treated with the PME algorithm [27] (real space cutoff 1 nm, FFT grid spacing 0.18 nm). The Lennard-Jones potentials were computed by using a cutoff length of 1.2 nm.

Free energy profiles for fullerenes were obtained using the metadynamics approach [28] and the plugin PLUMED [29]. Each of the metadynamics calculations was preceded with an equilibration run. A total of 1.5 µs of molecular dynamics was carried out. We chose a z-component (perpendicular to the membrane plane) of a vector connecting centers of masses of the membrane and the fullerene as a reaction coordinate (called *collective variable*) for the metadynamics simulations. The following parameters were used for metadynamics: the integration time step was decreased to 1 fs to improve stability of the systems, the time interval between the addition of two Gaussian functions was t = 500 fs, the Gaussian height w = 0.5 kJ/mol, and the Gaussian width d = 0.5 Å. Potential walls were applied to keep the values of the chosen collective variables in the area of a single membrane leaflet with the adjacent water layer, i.e. between −10 and 35 Å. Simulations were carried on until the convergence of the free energy surfaces was achieved after 45 ns. Additional 4 independent trajectories were obtained for each of the studied systems to calculate the average free energy profiles along with standard deviation. Sufficient sampling of the collective variables in metadynamics simulations was checked by plotting their progress along the simulation time (Figure S1) to confirm adequate sampling of the chosen reaction coordinate. All of the performed MD runs are listed in Table S2. Analysis of the obtained trajectories was done with standard Gromacs 4.5 utilities. Membrane properties calculations were made using GridMAT-MD [30]. Plots of molecular hydrophobic potential (MHP) for the membrane surfaces were built using PLATINUM software [31].

## Results and Discussion

### C_60_ fullerene rapidly penetrates into membrane

**Figure 2:**
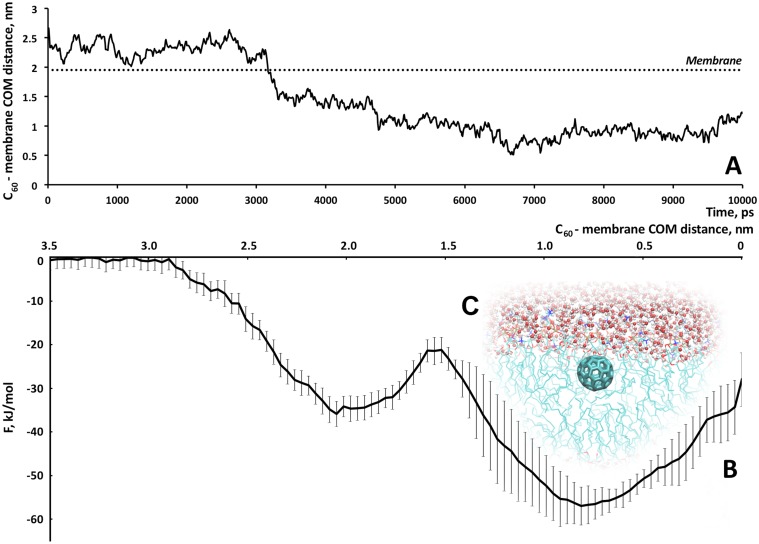
Characteristics of fullerene-membrane interactions. A. Distance between the center of mass (COM) of C_60_ and COM of the membrane. On the third nanosecond fullerene spontaneously “jump” into the membrane (the membrane surface is shown with the dot line). B. Free energy profile of the process of the C_60_ penetration into the model eukaryotic membrane. Potential wall at 30 Å is shown as the dotted line. C. Snapshot of the system with a single C_60_ molecule inside the membrane.

The equilibrium MD simulation of a single C_60_ performed in our study showed that during the first nanosecond of the MD trajectory the fullerene was adsorbed in the area of the lipid headgroups. However, in the third nanosecond a spontaneous dive into the tail region was observed (Figure 2A, C), which is consistent with [15], [16], [17]. Over the following almost 100 ns of the simulation the fullerene remains inside the membrane.

Table 1 represents a summary of previous studies of interactions of C_60_ and different membranes. According to this table we can conclude that the shape of the PMF profiles for the fullerene interacting with the membrane are quite similar. All methods, which were used to describe interactions between C_60_ and membranes, show a low minimum inside the membrane (near its center). Yet the details are different: some simulations show a small barrier for fullerene before entering the tail region. Also it’s important to notice that coarse-grain (CG) models underestimate free energy values for C_60_. Different methods of free energy calculations compromise energies: due to the small simulation time it is almost impossible to reach the thermodynamic equilibrium in such inhomogeneous systems as membranes.

**Table 1:** Comparison of available computational studies of the interaction of C_60_ with lipid bilayer.

#	*Force field*	*Membrane* *composition*	*Water* *model*	*Fullerene model*	*Extrema* *(PMF):* *Coordinate* *(nm)/PMF* *(kJ mol^−1^)*	*PMF calculation* *method*	*Reference*
1	GROMOS(Berger lipids)	DPPC	SPC	C_60_ is rigid body with a C-C bond length of1.46 Å. The LJ parameters for C-Care taken from Gromacs forcefield.	Min(1):2,7/−2,5;Max(1): 2,1/5;Min(2): 1,1/−24,8	The C_60_ moleculewas fixedatdifferent z-positions and theaverage force acting on it wascomputed during the simulation.Integration of the force gave PMF.	Qiao(2007)[17]
2	CHARMM27	DMPC	TIP3P	The carbon-carbon interaction in C_60_was described by a LJ potential withσ(C-C) 3.47 Å and ε(C-C) 0.275 kJmol^−1^.	Min(1):0,7/−83,7	Constraint force at different positionswas calculated for the fullerene. Integrationof the force gave PMF.	Bedrov(2008)[16]
3	MARTINI	(1) DOPC, (2) DPPC	MARTINI	Fullerene is 16 particles on a spherical surfacewith a diameter of 0.72 nm.All the particles are connected with anelastic network of bonds. The forceconstant for the bonds is 1250 kJ·mol^−1^·nm^−2^.	(1): Max(1):2,9/5; Min(1):1,2/−110. (2):Max(1):2,65/7;Min(1):0,9/−100	Umbrella Samplingmethod	Wong-Ekkabut(2008) [15]
4	MARTINI	DPPC	MARTINI	Fullerene is 20nonpolar particles.	Min(1):0/−183,5	Umbrella Samplingmethod	D’Rozario(2009) [18]
5	UA-OPLS	DMPC/cholesterol	TIP3P	DL_POLY 2.17 GUI interface was used togenerate nanoparticle intramolecular forces.	Min(1):0,9/−76	Constraintforce(CF) approachand thermodynamicintegration(TI)method	Fiedler(2010) [36]
6	CHARMM27	POPC	TIP3P	Parameters are taken from Bedrov(2008):σCC = 3.895 Å, εCC = 0.066kcal·mol^−1^	Max(1):2,2/2,1;Min(1):1,1/−36,8	Adaptive BiasingForce Method	Kraszewski(2011) [37]
7	GROMOS(Berger lipids)	DPPC	SPC/E	The lengths of the two types of C-Cbonds in fullerenes were set to 0,139 nmand 0,144 nm. LJ-parameters areσ(C-C) 3.47 Å and ε(C-C) 0.275 kJmol^−1^.	Min(1):2.0/−34;Max(1):1.6/13.5;Min(2):0.65/−57	Metadynamicssimulations	Our model

We have used the metadynamics approach to calculate the free energy profiles of the fullerenes interacting with the biomembrane, which, to the best of our knowledge, has not been applied to the fullerene-membrane systems before. So we have validated the method against the well-studied case of the C_60_ fullerene interacting with the membrane and came up with the conclusion that the metadynamics can be efficiently exploited in case of the C_3_ and D_3_ derivatives as well. In the figure 2B one can see that the fullerene overcomes the energy barrier of approximately 15 kJ/mol to pass into the hydrophobic region of the lipid tails. The global energy minimum for the C_60_ is located 0.7 nm behind the center of the membrane: if the C_60_ is moving closer to the center of the membrane its free energy is increasing by almost 30 kJ/mol. So, the C_60_ remains at a certain distance from the precise membrane center in the region of the hydrocarbon chains. To double-check this observation C_60_ was placed in the center of the membrane (between C-terminal atoms of lipid tails) and a 50 ns simulation was run. The fullerene in this simulation also migrated to the area located approximately 0,8 nm from the center of the membrane and remained there for the rest of the simulation. Absorption of a *single* C_60_ on the surface of membrane and its “jump” to the tails region cause no damage to the membrane (see Table 2). The above-mentioned results appear to be in a good agreement with the previously reported data serving as a good validation of the chosen model.

**Table 2:** Properties of the DPPC membrane in simulations with different amount of fullerenes.

*Membrane property*	*Pure DPPC*	*DPPC with one fullerene*	*DPPC with ten fullerenes*	
			70–100 ns of simulation	470–500 ns of simulation
Thickness, Å	33,7	32,0	38,7	42,2
Area per lipid, Å^2^	69	71	56	56,6
Lateral diffusion coefficient, 10^−7^ cm^2^/s	0,98	1,3	0,99	0,61
Membrane curvature	-	-	32	30,2

### Cluster of fullerenes causes changes in the physical properties of the membrane upon penetration

**Figure 3:**
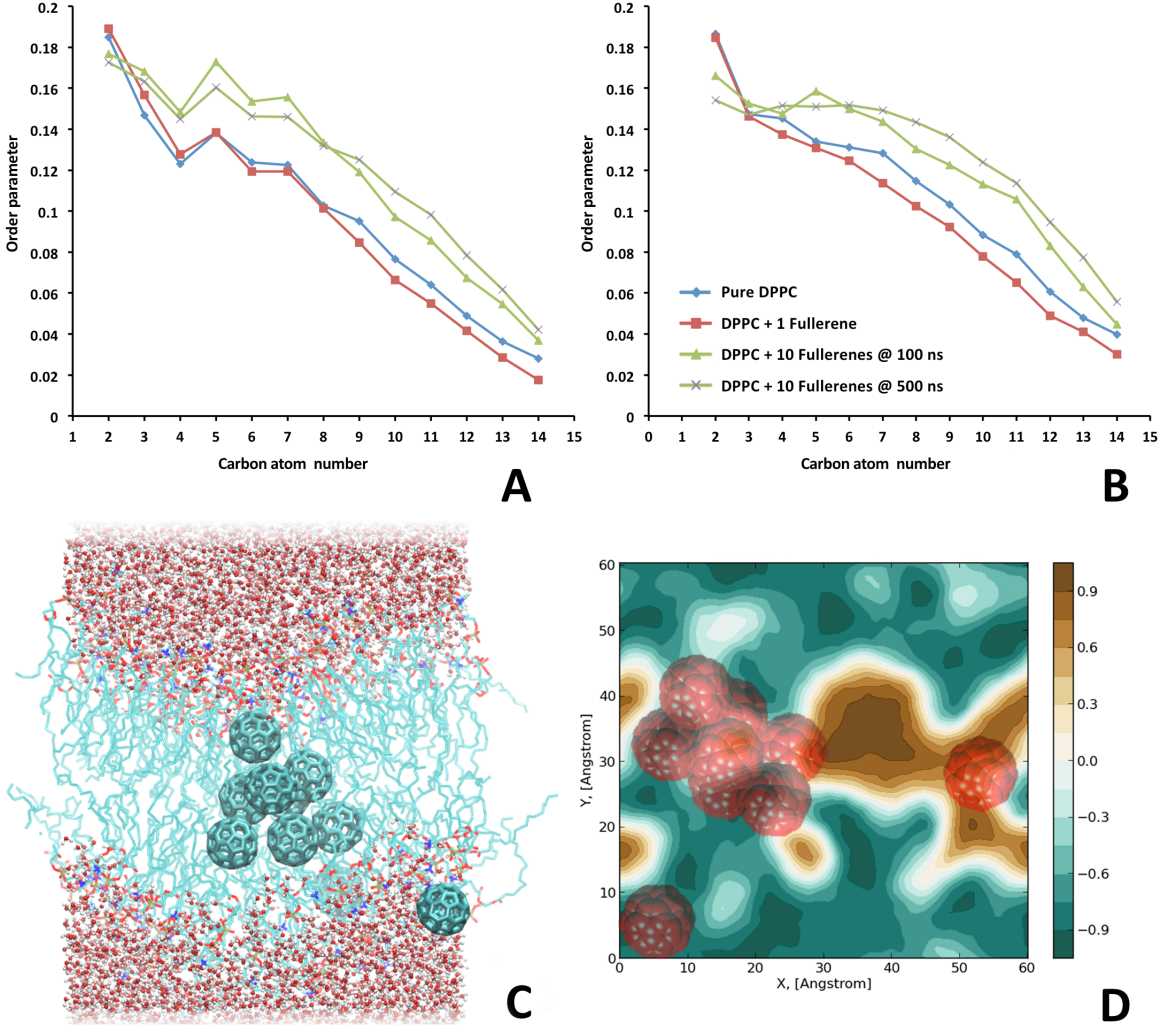
Order parameters, a snapshot of the final conformation of the system with ten C_60_ molecules and MHP map of the final conformation. A–B. Order parameters for lipid tails calculated from the equilibrium MD trajectories for the pure DPPC membrane, DPPC with one C_60_ molecule and DPPC with 10 C_60_ molecules: A–sn1-chain, B–sn-2 chain. C. Snapshot of the final conformation of the system with ten C_60_ molecules (totally nine fullerenes penetrated into the membrane). D. Molecular hydrophobic potential (MHP) map made for the upper surface of DPPC membrane. Carbone atoms of C_60_ are rendered with red VdW spheres.



In an aqueous medium fullerenes exist only in aggregates because of their hydrophobic nature. In our study we provide calculations of ten fullerenes and a DPPC membrane with heavy-atom resolution. To study the permeability of the membrane for a fullerene cluster which exists in real solutions, we assembled a system consisting of 10 fullerenes above the surface of the membrane. At the start of the simulation fullerenes did not contact with each other or with the membrane, but after a few nanoseconds, they aggregated and absorbed in the area of the lipid headgroups. The first fullerene from the cluster “jumped” inside the membrane after 3 ns. The rest of the fullerenes penetrated the membrane afterwards, so that eventually, after 100 ns, 9 of 10 fullerenes had dived into the membrane (Figure 3C). All of them stayed in the membrane during the next 400 ns of the simulation time. We did not observe their disaggregation during simulation time but according to [15] this happens in the microsecond scale. The most probable localization of fullerenes inside the membrane is around 0.5–1 nm from the center. Contrarily to the previously reported coarse-grained MD simulations [15], the membrane is deformed significantly by the addition of a large number of fullerenes: it curves (Figure 3C, average curvature radius calculated as in [32], Figure S2), the area per lipid head reduces from 69 to 56 Å^2^, on the contrary, thickness increases to 38,7 Å and up to 42 Å at the end of simulation. Such changes are connected to the formation of a fullerene cluster inside the membrane (Figure 3C) consisting of 8 fullerenes. MHP plots were made for both membrane sides of the two membrane conformations: at the beginning and at the end of the simulation (see SI: Figure S3). We observed that a large hydrophobic area had appeared on the membrane surface from the side where the fullerenes penetrated the membrane (Figure 3D). It means that when the fullerenes get inside the membrane a small pore appears with the hydrophobic lipid tails being exposed to the solvent. Lipids in this conformation are more vulnerable to ROS. The diffusion coefficients for DPPC molecules do not change upon addition of the fullerenes in the membrane and they are in a good agreement with the experimental data [33]. Pore or micelle formation is not observed though possibly due to insufficient simulation time. The order parameters for acyl chains S_CH_ (Figure 3A and 3B) for lipid chains sn-1 and sn-2 were calculated using the following equation:

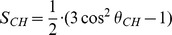



From these plots one can see that the order parameters do not change significantly when only one fullerene is inside the membrane while they modestly increase in the simulation with 9 fullerenes inside the membrane.

Our observations indicate that the addition of multiple C_60_ molecules does not cause a phase transition of the lipid bilayer but leads to its loosening and bending as well as to the formation of hydrophobic pores, what altogether can increase membrane susceptibility to ROS and explains toxicity of high concentrations of the C_60_, which was reported previously [34].

### C_3_ and D_3_ fullerene derivatives do not penetrate membrane

**Figure 4:**
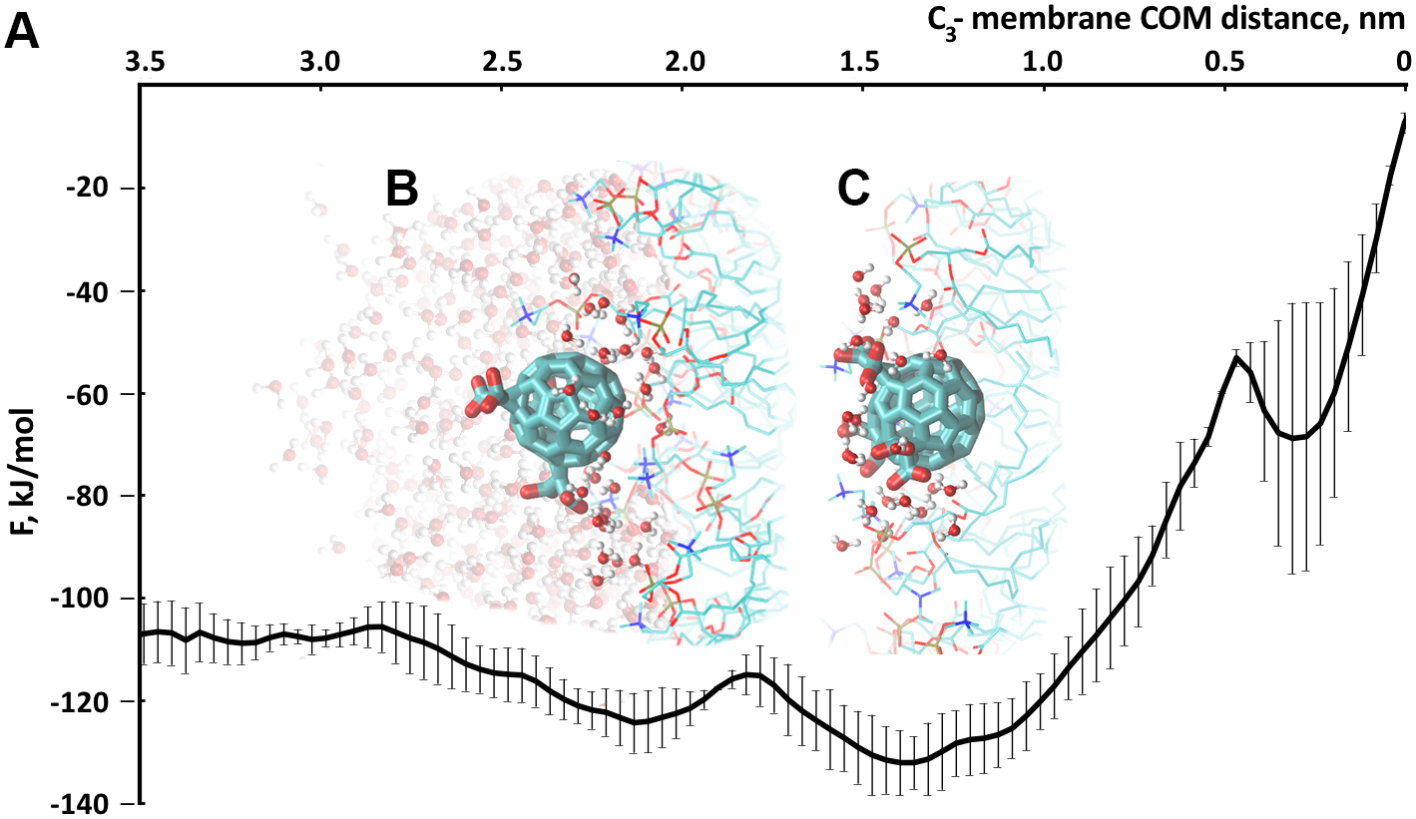
C_3_ stereoisomer of tris-malonic fullerene. A. Free energy profile of the process of the C_3_ penetration into the model eukaryotic membrane. B. Intermediate orientation of the C_3_ molecule adsorbed to the membrane with its solvent shell retained. C. Stable conformation (corresponding to the global energy minimum of the free energy profile) of C_3_ adsorbed to the membrane and established hydrophobic contact with the lipid tails region.

**Figure 5:**
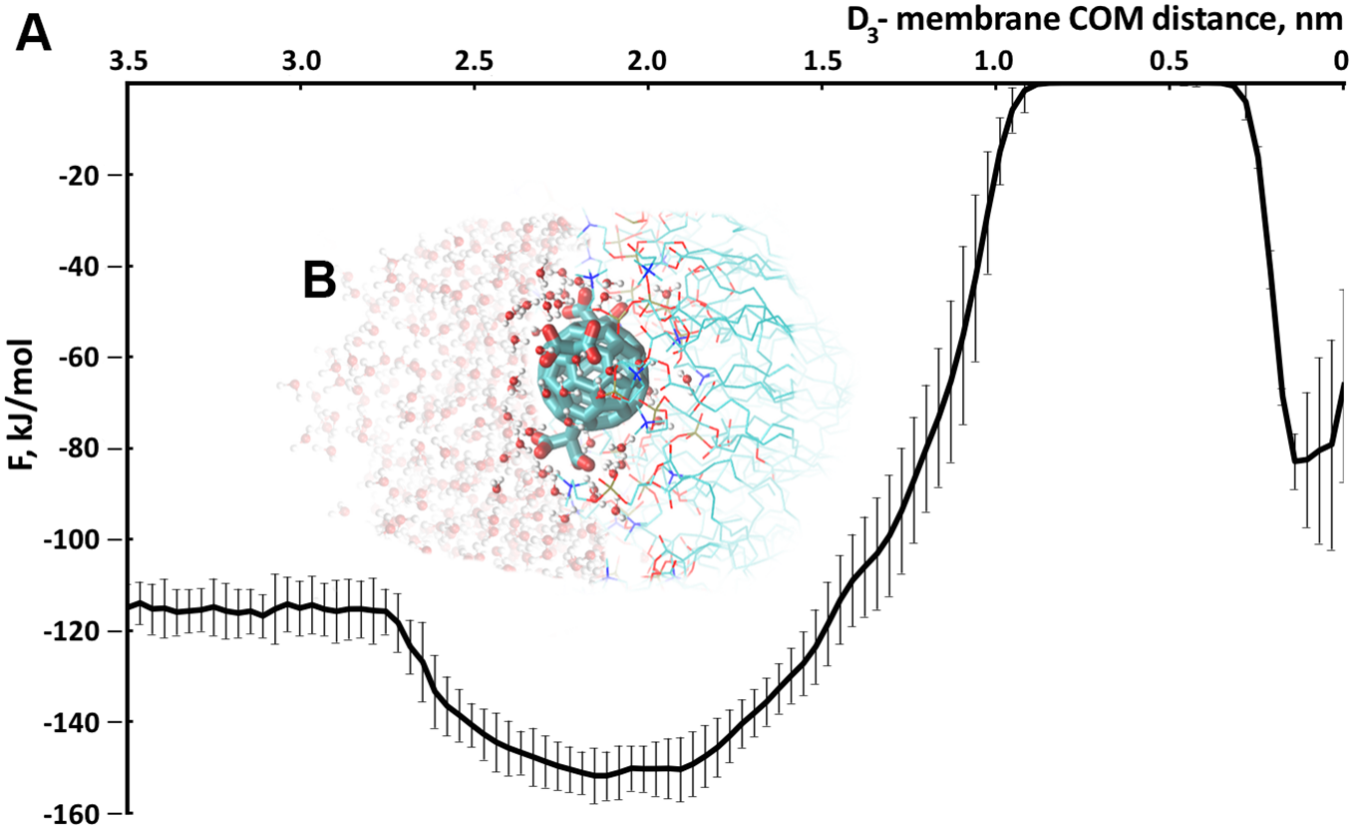
D_3_ stereoisomer of tris-malonic fullereneB A. Free energy profile of the process of the D_3_ penetration into the model eukaryotic membrane. B. Orientation (corresponding to the global energy minimum of the free energy profile) of the D_3_ molecule adsorbed to the membrane.

We have carried out equilibrium MD simulations of the C_3_ and D_3_ fullerenes interacting with the membrane along with metadynamics simulations in order to obtain a complete picture of molecular and energetical details of the membrane insertion processes for the C_3_ and D_3_ variants (Figure 4, 5).

The fully deprotonated forms of both studied fullerene derivatives should be the most probable species in aqueous environment (pK_a 1_ = 2.3 and pK_a 2_ = 5.7 for malonic acid in water). This seems to be no longer the case in the membrane hydrophobic region, where pK_a_s of acidic groups shift towards higher values. Higher pK_a_s values should favor protonated species and reduce thus the free energy cost for the translocation of the C_3_/D_3_ to the region of the lipid tails. On the other hand in the region of the zwitterionic lipid head groups pK_a_ of malonyl groups depends on the interplay of the local interactions of the fullerene derivatives with phosphate or choline groups. However, we assumed that these effects play secondary role at the stage of the C_3_/D_3_ absorption to the bilayer and performed simulations of the fully ionized C_3_/D_3_ species (charged −6) only.

In the equilibrium simulation with C_3_, the C_3_ molecule adsorbed to the membrane surface in two steps: in the beginning, it was localized at a distance of 2 nm from the COM of the membrane and retained its solvent shell (which consisted of 40 water molecules) as seen in Figure 4 and, later, it moved closer to the COM of the membrane by approximately 0.5 nm and established a hydrophobic contact with the lipid hydrocarbon tails as well as ionic bonds with the lipid headgroups (Figure 4). This notable behavior of the C_3_ derivative takes root in its stereochemistry–the molecule has two hemispheres: one is hydrophobic and the opposite carries three malonic acid residues and thus is hydrophilic (Figure 1C). We calculated the orientation of C_3_ molecule in respect to the membrane normal along the equilibrium trajectory of the C_3_ absorption to the membrane surface. Since the absorption process was completed after 25 ns, the C_3_ upheld a specific orientation with its hydrophobic hemisphere pointing to the middle of the membrane and the malonic groups interacting with the head groups of the lipids remained accessible to the solvent (Figure S4). Energetically, this orientation of the C_3_ corresponds to the global minimum of the free energy profile.

For both the malonic derivatives of C_60_ we also calculated their solvent accessible surface area (SAS). Upon the absorption of C_3_ to the membrane the SAS of C_3_ decreased significantly (Figure S5). On the other hand, the SAS of D_3_ remained almost the same during the whole simulation time, what indicates the lack of a close hydrophobic contact with the lipid tails in this case.

The observed orientation of C_3_ upon its absorption to the membrane surface appears to play a crucial role in efficient scavenging of ROS and radical protection: It resides not inside the membrane but near the lipid tail region, preventing chain peroxidation of the lipids. The isomeric form of C_3_–D_3_– does not have [35] such high cytoprotective properties since its three malonic acid residues cover the fullerene surface symmetrically (Figure 1B) and D_3_ only adsorbs efficiently to the region of the lipid headgroups where the distance from the COM of the membrane equals 2 nm and retains its solvent shell (see Figure 5C).

Neither the C_3_ nor the D_3_ stereoisomers penetrate the membrane because of the large free energy penalty of being in the middle of the membrane for these molecules (Figure 4A and 5A).

## Conclusions

The processes of penetration and accumulation of C_60_ fullerene, its cluster and the two derivatives of C_60_ in lipid bilayers were studied using equilibrium MD and metadynamics approach. Absorption of all of the studied fullerene species on the DPPC membrane occurs within the first nanoseconds. C_60_ fullerene (a single molecule and a cluster of ten molecules) spontaneously penetrates the membrane and remains inside of it throughout the whole simulation time. After penetration into the eukaryotic membrane, C_60_ stays at a distance of 0.7–0.8 nm from the center of the membrane. A cluster of fullerenes deforms the membrane causing its curvature and formation of hydrophobic regions, which presumably make the membrane more susceptible to the ROS attacks.

The studied tris-malonic derivatives of the C_60_ fullerene do not penetrate membrane but rather are accumulated at its surface. However, during adsorption to the membrane the C_3_, in contrast to the D_3_, appears to be deeply buried in the area of the lipid headgroups of the membrane forming specific hydrophobic contacts with the hydrocarbon tails of the lipids along with the ionic interactions with the head region and hydrophilic contacts with the solvent. This energetically favorable orientation of the C_3_ derivative, as we assume, accounts for its high ROS scavenging activity comparing to the D_3_.

## Supporting Information

Figure S1 **Progress of the collective variable during the C_60_, C_3_ and D_3_ metadynamics simulations.**
(TIF)

Figure S2 **Membrane curvature in xz and yz directions.**
(TIF)

Figure S3 **Molecular hydrophobic potential map built for lower (A, C) and upper (B, D) sides: A, B–in the beginning of simulation C, D–in the end of simulation.** Fullerenes are shown as red spheres.
(TIF)

Figure S4 **Orientation of C_3_ molecule during to the bilayer normal during equilibrium MD simulation.**
(TIF)

Figure S5 **SAS of C_3_ and D_3_ molecules during equilibrium MD simulation.**
(TIF)

Table S1 **Charges for C_3_ and D_3_ fullerenes.**
(DOCX)

Table S2 **The summary of the performed MD simulations.**
(DOCX)

Text S1 **Creating Models of C_3_ and D_3._**
(DOCX)
