# Supplementary figures and images for: Comparative Computational Study of Interaction of C60-Fullerene and Tris-Malonyl-C60-Fullerene Isomers with Lipid Bilayer: Relation to Their Antioxidant Effect
Source: PLoS One. 2014 Jul 14;9(7):e102487. doi: 10.1371/journal.pone.0102487 (PMC4097404; doi:10.1371/journal.pone.0102487)

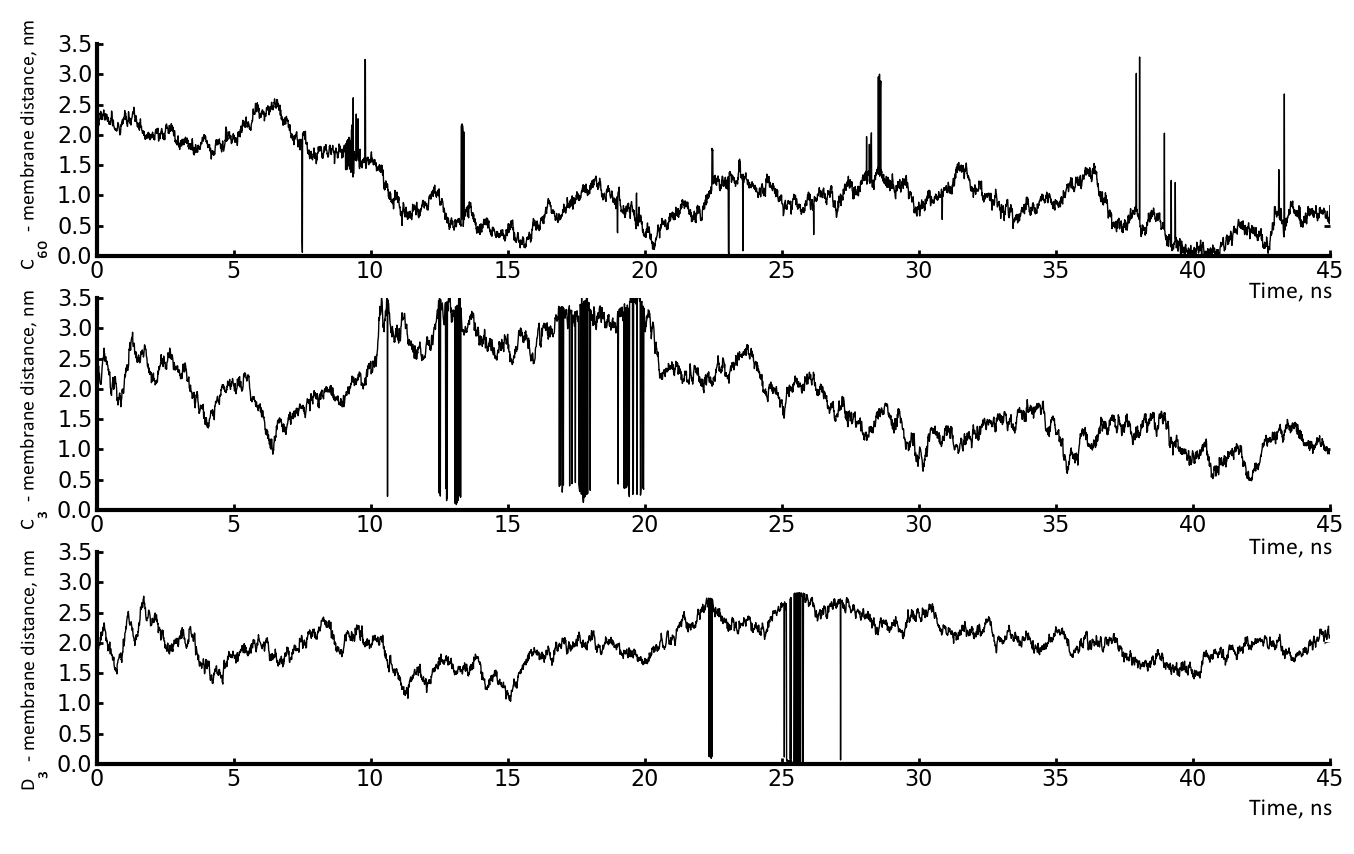

Supplement: Figure S1 — Progress of the collective variable during the C60, C3 and D3 metadynamics simulations. (TIF) [file pone.0102487.s001.tif]

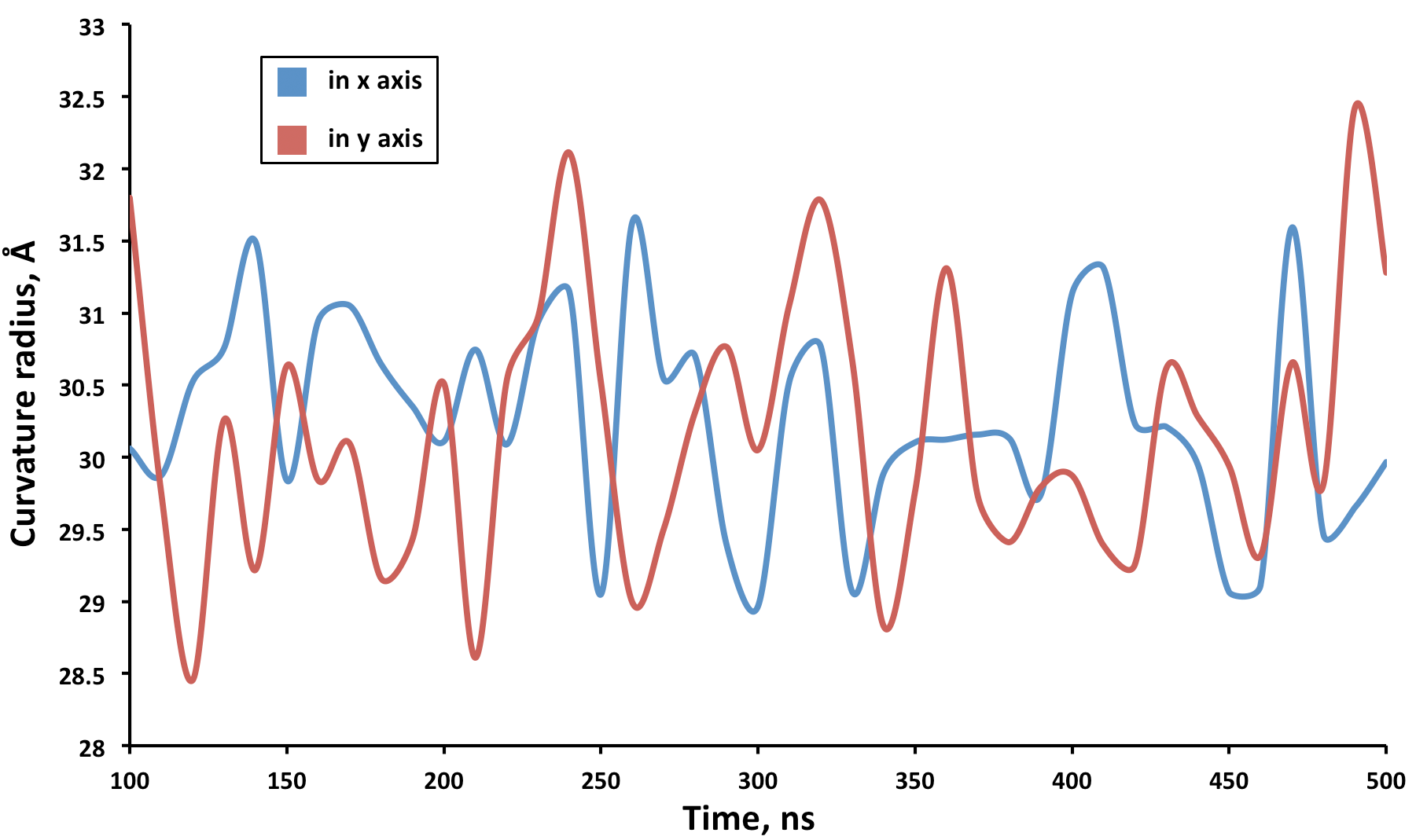

Supplement: Figure S2 — Membrane curvature in xz and yz directions. (TIF) [file pone.0102487.s002.tif]

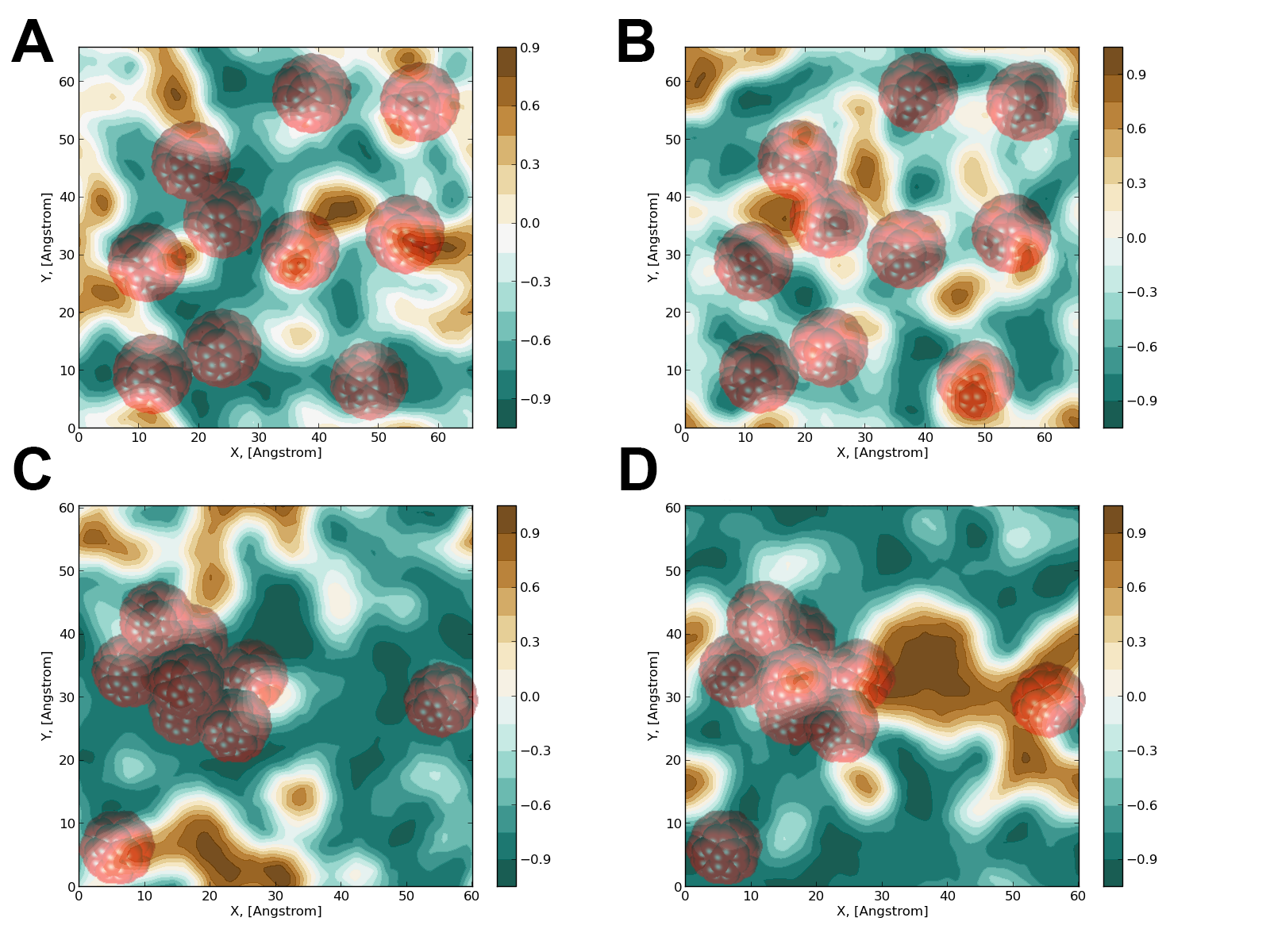

Supplement: Figure S3 — Molecular hydrophobic potential map built for lower (A, C) and upper (B, D) sides: A, B–in the beginning of simulation C, D–in the end of simulation. Fullerenes are shown as red spheres. (TIF) [file pone.0102487.s003.tif]

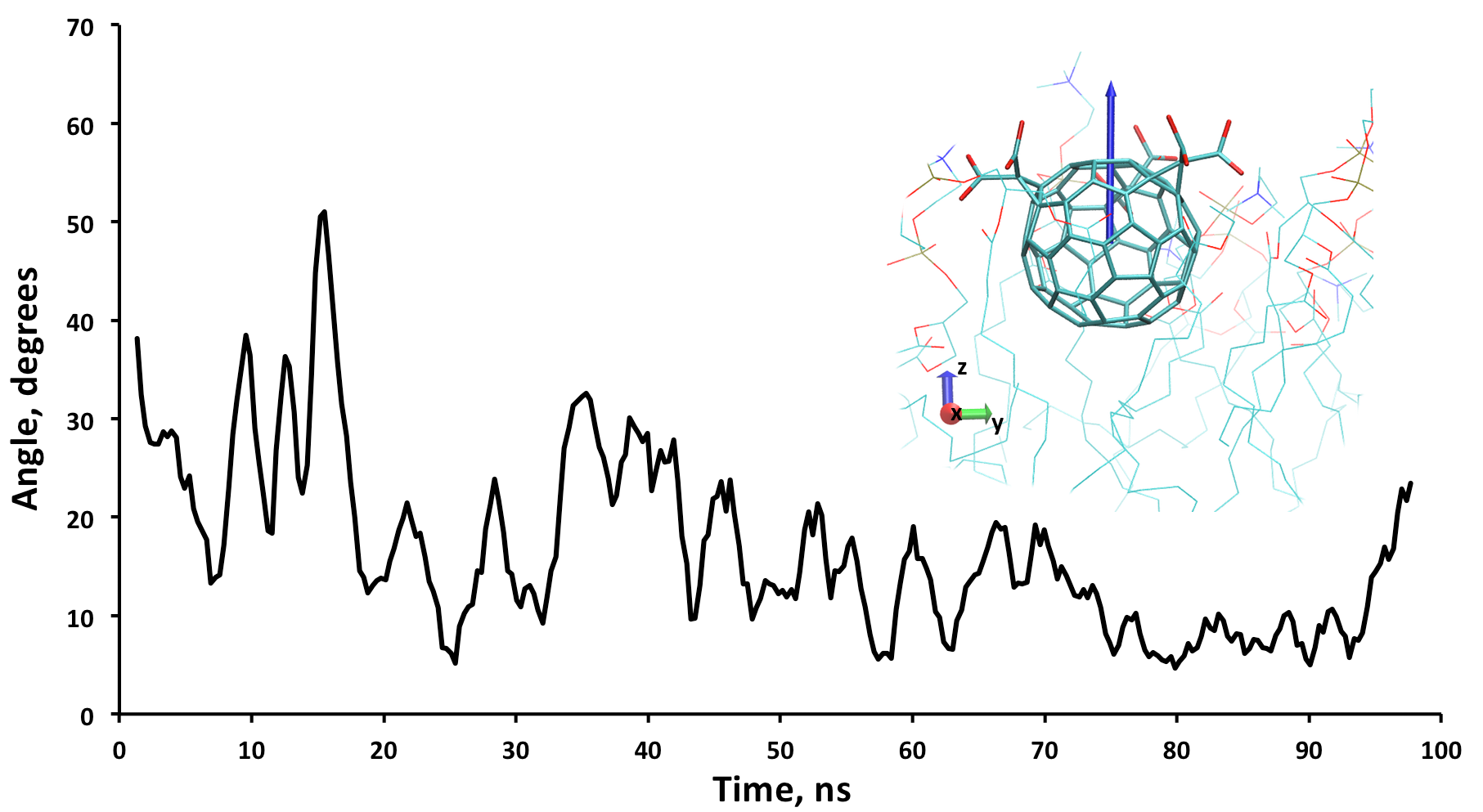

Supplement: Figure S4 — Orientation of C3 molecule during to the bilayer normal during equilibrium MD simulation. (TIF) [file pone.0102487.s004.tif]

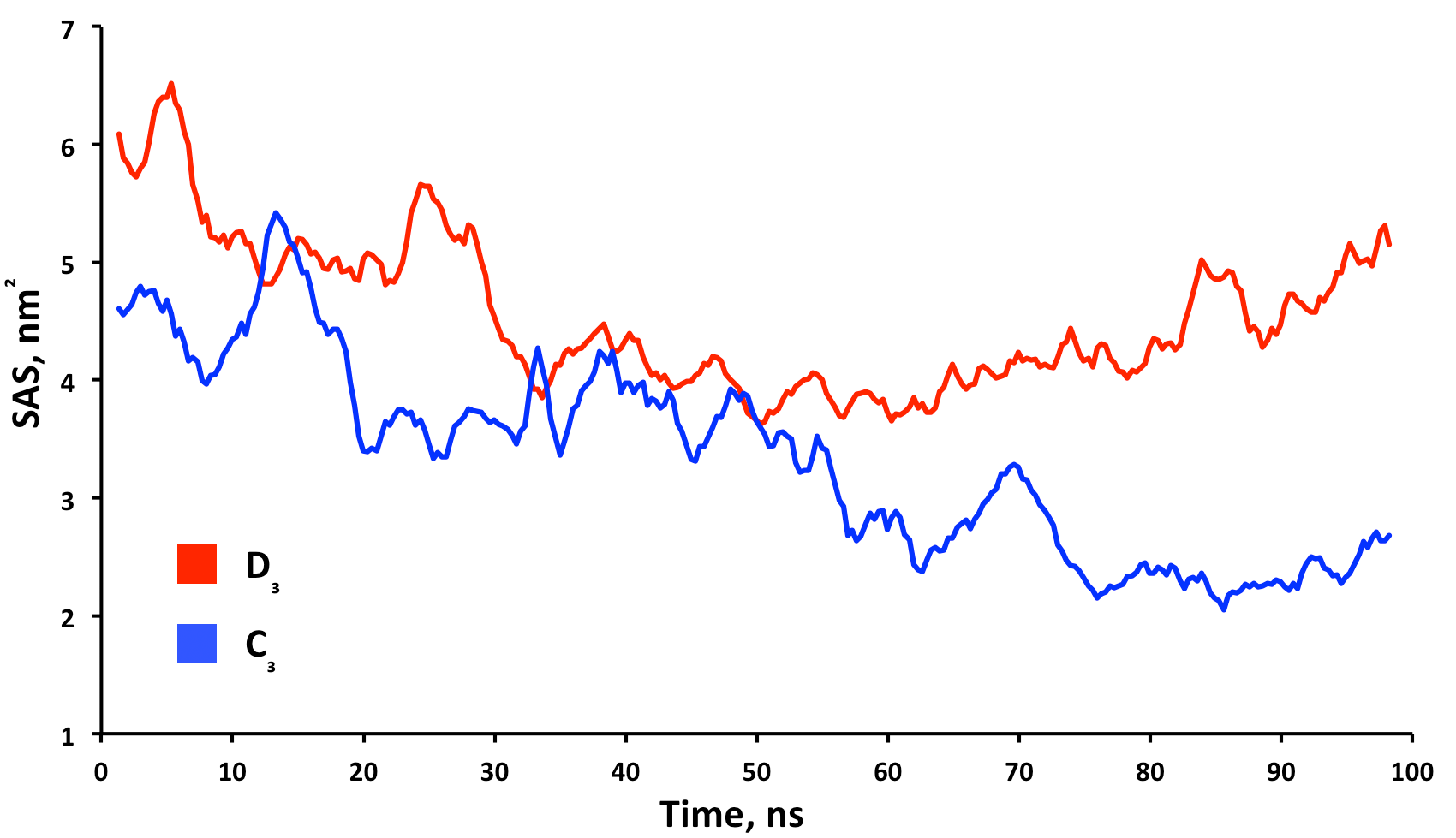

Supplement: Figure S5 — SAS of C3 and D3 molecules during equilibrium MD simulation. (TIF) [file pone.0102487.s005.tif]
