# Supplementary material for: Comparative Computational Study of Interaction of C60-Fullerene and Tris-Malonyl-C60-Fullerene Isomers with Lipid Bilayer: Relation to Their Antioxidant Effect
Source: PLoS One. 2014 Jul 14;9(7):e102487. doi: 10.1371/journal.pone.0102487 (PMC4097404; doi:10.1371/journal.pone.0102487)
Supplement: Table S1 — Charges for C3 and D3 fullerenes. (DOCX) [file pone.0102487.s006.docx]

| ***Atom №*** | ***Atom name*** | ***Partial charge*** | ***Atom №*** | ***Atom name*** | ***Parial charge*** | ***Atom №*** | ***Atom name*** | ***Partial charge*** |
| --- | --- | --- | --- | --- | --- | --- | --- | --- |
| 1 | CF1 | 0 | 28 | CF28 | 0 | 55 | CF55 | 0 |
| 2 | CF2 | 0 | 29 | CF29 | 0 | 56 | CF56 | 0 |
| 3 | CF3 | 0 | 30 | CF30 | 0 | 57 | CF57 | 0 |
| 4 | CF4 | 0 | 31 | CF31 | 0 | 58 | CF58 | 0 |
| 5 | CF5 | 0 | 32 | CF32 | 0 | 59 | CF59 | 0 |
| 6 | CF6 | 0 | 33 | CF33 | 0 | 60 | CF60 | 0 |
| 7 | CF7 | 0 | 34 | CF34 | 0 | 61 | C1 | 0,098 |
| 8 | CF8 | 0 | 35 | CF35 | 0 | 62 | C2 | 0,271 |
| 9 | CF9 | 0 | 36 | CF36 | 0 | 63 | C3 | 0,271 |
| 10 | CF10 | 0 | 37 | CF37 | 0 | 64 | C4 | 0,098 |
| 11 | CF11 | 0 | 38 | CF38 | 0 | 65 | C5 | 0,271 |
| 12 | CF12 | 0 | 39 | CF39 | 0 | 66 | C6 | 0,271 |
| 13 | CF13 | 0 | 40 | CF40 | 0 | 67 | C7 | 0,098 |
| 14 | CF14 | 0 | 41 | CF41 | 0 | 68 | C8 | 0,271 |
| 15 | CF15 | 0 | 42 | CF42 | 0 | 69 | C9 | 0,271 |
| 16 | CF16 | 0 | 43 | CF43 | 0 | 70 | O1 | -0,66 |
| 17 | CF17 | 0 | 44 | CF44 | 0 | 71 | O3 | -0,66 |
| 18 | CF18 | 0 | 45 | CF45 | 0 | 72 | O2 | -0,66 |
| 19 | CF19 | 0 | 46 | CF46 | 0 | 73 | O4 | -0,66 |
| 20 | CF20 | 0 | 47 | CF47 | 0 | 74 | O7 | -0,66 |
| 21 | CF21 | 0 | 48 | CF48 | 0 | 75 | O8 | -0,66 |
| 22 | CF22 | 0 | 49 | CF49 | 0 | 76 | O6 | -0,66 |
| 23 | CF23 | 0 | 50 | CF50 | 0 | 77 | O5 | -0,66 |
| 24 | CF24 | 0 | 51 | CF51 | 0 | 78 | O9 | -0,66 |
| 25 | CF25 | 0 | 52 | CF52 | 0 | 79 | O10 | -0,66 |
| 26 | CF26 | 0 | 53 | CF53 | 0 | 80 | O11 | -0,66 |
| 27 | CF27 | 0 | 54 | CF54 | 0 | 81 | O12 | -0,66 |
