# Supplementary material for: Comparative Computational Study of Interaction of C60-Fullerene and Tris-Malonyl-C60-Fullerene Isomers with Lipid Bilayer: Relation to Their Antioxidant Effect
Source: PLoS One. 2014 Jul 14;9(7):e102487. doi: 10.1371/journal.pone.0102487 (PMC4097404; doi:10.1371/journal.pone.0102487)
Supplement: Table S2 — The summary of the performed MD simulations. (DOCX) [file pone.0102487.s007.docx]

| ***System*** | ***Trajectory length, ns*** | ***System size, atoms*** | ***Type of run*** |
| --- | --- | --- | --- |
| Pure hydrated membrane | 100 | 17353 | Equilibrium MD |
| 1 molecule of C_60_ | 100 | 17413 | Equilibrium MD started with C_60_ placed outside the membrane |
| 1 molecule of C_60_ | 50 | 17413 | Equilibrium MD started with C_60_ placed within the membrane |
| 10 molecules of C_60_ | 500 | 23854 | Equilibrium MD |
| 1 molecule of C_60_ | 25 × 5 | 23938 | Metadynamics |
| 1 molecule of C_3_ | 100 | 23966 | Equilibrium MD |
| 1 molecule of C_3_ | 45 × 5 | 23966 | Metadynamics |
| 1 molecule of D_3_ | 100 | 16924 | Equilibrium MD |
| 1 molecule of D_3_ | 45 × 5 | 16924 | Metadynamics |
