# Supplementary material for: Comparative Computational Study of Interaction of C60-Fullerene and Tris-Malonyl-C60-Fullerene Isomers with Lipid Bilayer: Relation to Their Antioxidant Effect
Source: PLoS One. 2014 Jul 14;9(7):e102487. doi: 10.1371/journal.pone.0102487 (PMC4097404; doi:10.1371/journal.pone.0102487)
Supplement: Text S1 — Creating Models of C3 and D3. (DOCX) [file pone.0102487.s008.docx]

**Creating Models of C_3_ and D_3_**

C_3_ and D_3_ models were built in the program HyperChem. The charges were calculated in the GAMESS suite using *ab initio* Hartree-Fock method with 6-31G** basis set (prefaced by the geometry optimization of the molecules at the same level of theory). Parameters for LJ interactions for malonic acid were taken from Gromacs topologies. Both molecules are negatively charged in water due to the pKa values of 2.3 and 5.7 for malonic acid in water (Table S1).
